# Psychometric properties of an Arabic translation of the briefest version of the Zimbardo time perspective inventory (ZTPI-15)

**DOI:** 10.1186/s12888-023-04815-8

**Published:** 2023-05-12

**Authors:** Feten Fekih-Romdhane, Abdallah Chahine, Mariam Mhanna, Christian Joseph El Zouki, Sahar Obeid, Souheil Hallit

**Affiliations:** 1 The Tunisian Center of Early Intervention in Psychosis, Department of psychiatry “Ibn Omrane”, Razi hospital, 2010 Manouba, Tunisia; 2 Faculty of Medicine of Tunis, Tunis El Manar University, Tunis, Tunisia; 3 School of Medicine and Medical Sciences, Holy Spirit University of Kaslik, P.O. Box 446, Jounieh, Lebanon; 4 Social and Education Sciences Department, School of Arts and Sciences, Lebanese American University, Jbeil, Lebanon; 5 Psychology Department, College of Humanities, Effat University, Jeddah, 21478 Saudi Arabia; 6 Applied Science Research Center, Applied Science Private University, Amman, Jordan; 7 Research Department, Psychiatric Hospital of the Cross, Jal Eddib, Lebanon

**Keywords:** Time perspective, ZTPI-15, Psychometric properties, Validation, Arabic

## Abstract

**Background:**

Self-perceived temporal perspectives has been shown to vary across cultures. Although cross-cultural differences may be blurred by the globalization, accelerated pace-of-life worldwide and spread of multitasking; the way Arab individuals deal with time has its specificities. However, research in this area is scant in the Arab world. One of the main reasons for this scarcity of research is the lack of psychometrically sound and convenient-to-use measures. We aimed to examine the psychometric properties of an Arabic translation of the briefest version of the Zimbardo Time Perspective Inventory (ZPTI-15).

**Methods:**

A sample of community Arabic-speaking Adults from Lebanon (N = 423, 68.6% females, mean age 29.19 ± 12.54 years) were administered the Arabic ZPTI-15. The forward and backward translation method was adopted.

**Results:**

Confirmatory Factor Analyses (CFA) revealed that the five-factor model exhibited a good fit to the data. The five ZTPI-15 subscales yielded a McDonald’s omega ranging from 0.43 to 0.84. Multi-group CFA ascertained the invariance of the Arabic ZTPI-15 across gender at the configural, metric, and scalar levels. Our findings support divergent validity of the scale by showing positive correlations between past negative, present fatalistic, present hedonistic dimensions, and psychological distress; as well as negative correlations between past positive, future focused dimensions, and distress.

**Conclusion:**

The Arabic ZTPI-15 is easy-to-use, valid, reliable, and will hopefully enable the conduction of future research in the field to purposively provide comprehensive insight into the time perspective patterns and correlates in Arab countries, and the broad Arabic-speaking community globally.

**Supplementary Information:**

The online version contains supplementary material available at 10.1186/s12888-023-04815-8.

## Introduction

Time perspective is a psychological construct that refers to “the often non-conscious process whereby the continual flows of personal and social experiences are assigned to temporal categories, or time frames, that help give order, coherence, and meaning to those events” ([1], p. 1271). In other words, it represents the different ways in which the flow of one’s personal experiences is partitioned into temporal categories or time zones [2]. During the last decades, time perspective has gained a growing research interest due to its potential utility in understanding how this construct relates to a wide range of psychopathology and human behavior. Previous studies have, for example, documented significant and relevant associations between time perspective and depression, anxiety symptoms [1, 3, 4], aggression [1], risky driving [5], substance use [6, 7], maladaptive personality traits [8], self-esteem [4], self-regulation [9], self-efficacy [10], coping behavior [11], subjective well-being [12], life satisfaction [13], happiness [14], and academic achievement [15]. For instance, boredom and sadness predicted a perceived slowing down of the passage of time [16]. Additionally, depressed individuals overestimate the likelihood of negative events in their future, undervalue the prospect of positive events, and have dark expectations for the future [17]. As for alcohol use disorder, a rational decision to engage in heavy drinking may depend on individuals thinking about, or placing greater value on, the positive short-term effects more than they think about, or ‘dis-value’, the negative longer-term effects—i.e. having more future-orientated time perspective [18]. On the other hand, Konowalczyk et al. revealed that adolescents who had a positive perspective exercised more and had more self-esteem and, as a result, did not seek to engage in risky behaviors [19].

With the raise of awareness about the relevance of temporal psychology in the fields of clinical psychology and psychiatry, a number of measures have been developed to assess this entity, including the Adolescent and Adult Time Inventory–Time Attitudes Scale [20, 21], and the Temporal Focus Scale [22]. Nevertheless, these are rather narrowly focused measures, exclusively reflecting affect and cognitions, respectively; which may limit investigations and understandings of the time perspective construct. One of the first developed and largely used research instruments designed to assess broader facets of the time perspective construct (i.e., affect, cognition, and behavior) is the Zimbardo Time Perspective Inventory (ZTPI; [1]). The original ZTPI is composed of 56 items and five dimensions, i.e. (1) past positive (PP), which evaluates happiness and warmth regarding past events; (2) past negative (PN), which evaluates an overall sense of pessimism about past events; (3) present fatalistic (PF), which relates to the feeling of being powerlessness over life and the fate as determined by uncontrolled external force; (4) present hedonistic (PH), which describes the feelings of risk taking, pleasure, and enjoyment of life; and (5) future (F), which describes plans of achieving long-term outcomes and goals.

Apart from its utility in community research, the ZTPI has recently been recognized as a valuable diagnostic, preventive and therapeutic tool in clinical practice [23]. For these reasons, the original version of the ZTPI has been translated to different languages and validated in different countries, including France [24], Spain [25], Ukraine [26], Russia [27], Portugal [28], Brazil [29], Greece [4], the Netherlands [30], Serbia [31], Sweden [32], Mexico [33], Japan [34], and Algeria [35]. All these versions supported the validity and reliability of the ZTPI to assess individual differences in five time perspective categories. Its invariance across many countries and cultures has also been demonstrated [36]. At the same time, the original version has been largely criticized for its numerous limitations. Indeed, due to its length, the original ZTPI may be challenging to administer for both the clinician (or researcher) and the examined individual, especially where resources and time are a concern. This has resulted in the use of the incomplete scale (only three-time perspective or fewer instead of all five dimensions, e.g., [5, 37–40]); which has led in turn to missing information. In addition, the original 56-item ZTPI has presented factorial validity issues [41]. Therefore, several brief versions have been designed with the purpose of overcoming these gaps (36-item [36], 25-item [42], 20-item [43], 17-item [44], and 15-item [45, 46]), by removing the items with the lowest factor loadings and reducing the number of estimated parameters [41]. The ZTPI-15 has been considered as “the most comprehensive validation of a short ZTPI” [41]. To our knowledge, the 56-item ZTPI is the only version that has previously been validated in the Arabic language in 2009 among Algerian university students [35]; and no Arabic brief versions exist so far.

### The present validation study

The subjective self-perceived temporal perspectives or time duration and synchrony has been shown to vary across cultures [47]. For instance, people in “clock-time” cultural contexts strictly adhere to punctuality and schedules, while people in “event-time” cultural backgrounds tend to rely more on the natural flow of social events [48]. Although these cross-cultural differences may possibly be blurred by the globalization, accelerated pace-of-life worldwide and spread of multitasking; the way Arab individuals deal with time, perceive pace-of-life, or view the present, past, and future still has its specificities. Arab people often deal with time based on God willing (“inshallah”), lack of punctuality (failure to stick to appointments between family and friends), perceived shortage of time (“I do not have time”), huge time waste (e.g., customers are most of the time asked to wait or come back next day in governmental institutions), and a highly required flexibility (e.g., people may spend hours waiting for an appointment or a late meeting) [49]. To highlight the perception of time perspective in Arab countries, studies showed that the current time of economic crises in many Arab countries, those with insecure economic situations incline to move away from a focus on the future of saving and investing toward a more pragmatic routine, living each day as it comes [50]. Another study [49] revealed that Arab people are mostly oriented by the present-hedonistic and future and are not really oriented by fatalistic issues. In addition, the same study shows that there are significant differences among the five dimensions of TP and no balance in TP is recognized as a main prerequisite for psychological health, happiness, satisfaction in life, self-esteem and general wellbeing.

However, it is worth noting that research in this area is scant in the Arab world. We could identify only two studies focusing on time perspective, the Algerian validation study mentioned above, and another study that used the original 56-item English version of ZPTI among 122 community adults living in five Arab countries (Bahrain, Egypt, Iraq, Jordan and Sudan) [49]. One of the main reasons for this scarcity of research is the lack of psychometrically sound and convenient-to-use measures.

In this context, we aimed to examine the psychometric properties of an Arabic translation of the briefest version of the ZPTI (i.e. the ZPTI-15) in terms of factor structure, internal consistency, discriminant validity as well as gender invariance in a sample of community Arabic-speaking Adults from Lebanon. We expected that the Arabic version will confirm the five-factor structure of the scale, and will show adequate internal consistency reliability, appropriate discriminant validity, and measurement invariance across gender groups.

## Methods

### Participants

Four hundred twenty-three participants participated in this study, with a mean age of 29.19 ± 12.54 years (min = 18; max = 85) and 68.6% females. Other descriptive statistics of the sample can be found in Table 1.

**Table 1:** Sociodemographic and other characteristics of the sample (N = 423)

Variable	N (%)
Sex	
Male	133 (31.4%)
Female	290 (68.6%)
Marital status	
Single	315 (74.5%)
Married	108 (25.5%)
Education level	
Secondary or less	22 (5.2%)
University	401 (94.8%)
	**Mean ± SD**
Age (years)	29.19 ± 12.54
Household crowding index (persons/room)	0.98 ± 0.44

### Measures

#### The ZTPI-15

The ZPTI has originally been developed in its 56-item version by Zimbardo and Boyd [1], and shortened later to the 15-item version in English, Slovak and Czech languages by two groups of researchers [45, 46]. In this study, we translated and validated the English version of the ZPTI-15 [45]. The forward and backward translation method was applied to the ZTPI scale. A common procedure of back-translation was followed in the present study, in which a text is translated from a source into a target language, and then independently back-translated into the source language by a second interpreter. Therefore, the English version of the ZTPI-15 was translated to Arabic (Appendix 1) by a Lebanese translator who was completely unrelated to the study. Afterwards, a Lebanese psychologist with a full working proficiency in English, translated the Arabic version back to English. To evaluate the accuracy of the translation, the initial and back-translated English versions were compared [51, 52]; and any inconsistencies were detected and eliminated by a committee composed of the research team and the two translators. A pilot study was done on 20 participants to make sure that the questions are well understood; no changes were done afterwards [53].

Each item of the scale is scored on a five-point Likert scale, ranging from 1 (less important) to 5 (very important), with higher scores indicating a stronger applicability of the statement applies to the respondent.

#### Depression anxiety stress Scale-8 (DASS-8)

The DASS-8 contains eight items divided into three subscales: stress (two items), anxiety (three items), and depression (three items). The DASS-8 has been developed and validated in the Arabic language [54]. In this study, the subscales yielded the following McDonald’s omega values : stress (0.71), anxiety (0.84), and depression (0.80).

#### Demographics

Participants were asked to provide their demographic details consisting of age, sex, highest educational attainment, marital status and the Household Crowding Index (HCI); the latter reflecting the socioeconomic status of the family [55], is the ratio of the number of persons living in the house over the number of rooms in it (excluding the kitchen and the bathrooms).

### Procedures

The “Snowball Sampling” technique using Google Forms was carried out to collect the necessary data for the investigation, between August to November 2022. The project was advertised on social media and included an estimated duration. Indeed, participants were first invited to complete the questionnaire which link was initially distributed via social media applications such as ‘WhatsApp’, ‘Instagram’ and ‘Facebook’, and then asked to share it with their acquaintances, friends and/or family members.

Inclusion criteria for participation included being of a resident and citizen of Lebanon of adult age (≥ 18 years). Excluded were those who refused to fill out the questionnaire. Internet protocol (IP) addresses were examined to ensure that no participant took the survey more than once. After providing digital informed consent, participants were asked to complete the instruments described above, which were presented in a pre-randomised order to control for order effects. The survey was anonymous and participants completed the survey voluntarily and without remuneration. Before proceeding with the questionnaire, participants were informed of the purpose of the study, assured of the anonymity of their participation and provided with a virtual informed consent form via ‘Google Forms’. The latter had to be ‘signed’, after reading, by clicking the box ‘Yes, I acknowledge having read the above-mentioned information and I agree to participate in this study voluntarily and without any pressure’ to which the answer is required in order to continue with the self-administration. Participants had the right to accept or refuse to respond and no financial compensation was provided in exchange for their submission [56].

### Analytic strategy

#### Confirmatory factor analysis

There were no missing responses in the dataset. We used data from the total sample to conduct a CFA using the SPSS AMOS v.26 software. The minimum sample size to conduct a confirmatory factor analysis ranges from 3 to 20 times the number of the scale’s variables [57]. Therefore, we assumed a minimum sample of 240 participants needed to have enough statistical power based on a ratio of 20 participants per one item of the scale, which was exceeded in our sample. Our intention was to test the original model of the ZTPI scores (i.e., five-factor model). Parameter estimates were obtained using the maximum likelihood method and fit indices. Additionally, evidence of convergent validity was assessed in this subsample using the average variance extracted (AVE) values of ≥ 0.50 considered adequate [58] and meaning that a latent variable is able to explain more than half of the variance of its indicators on average (i.e., items converge into a uniform construct).

#### Gender invariance

To examine gender invariance of ZTPI scores, we conducted multi-group CFA [59] using the total sample. Measurement invariance was assessed at the configural, metric, and scalar levels [60]. Configural invariance implies that the latent scales variable(s) and the pattern of loadings of the latent variable(s) on indicators are similar across gender (i.e., the unconstrained latent model should fit the data well in both groups). Metric invariance implies that the magnitude of the loadings is similar across gender; this is tested by comparing two nested models consisting of a baseline model and an invariance model. Lastly, scalar invariance implies that both the item loadings and item intercepts are similar across gender and is examined using the same nested-model comparison strategy as with metric invariance [59]. Following the recommendations of Cheung and Rensvold (2002) [61] and Chen (2007) [59], we accepted ΔCFI ≤ 0.010 and ΔRMSEA ≤ 0.015 or ΔSRMR ≤ 0.010 (0.030 for factorial invariance) as evidence of invariance.

#### Further analyses

Composite reliability in both subsamples was assessed using McDonald’s (1970) ω, with values greater than 0.70 reflecting adequate composite reliability [62]. McDonald’s ω was selected as a measure of composite reliability because of known problems with the use of Cronbach’s α (e.g., [63]). To assess divergent validity, we examined bivariate correlations between ZTPI and mental health scores measured by DASS-8. Based on Cohen (1992) [64], values ≤ 0.10 were considered weak, ~ 0.30 were considered moderate, and ~ 0.50 were considered strong correlations.

## Results

### Confirmatory factor analysis of the ZTPI scale

CFA indicated that fit of the five-factor model of the ZTPI scale was acceptable: χ^2^/df = 242.17/80 = 3.03, RMSEA = 0.069 (90% CI 0.059, 0.079), SRMR = 0.070, CFI = 0.880, TLI = 0.842. When adding a correlation between residuals of items 6 and 7, the fit indices improved as follows: χ2/df = 190.70/79 = 2.41, RMSEA = 0.058 (90% CI 0.047, 0.068), SRMR = 0.064, CFI = 0.917, TLI = 0.890. The standardised estimates of factor loadings were all adequate (see Table 2). The convergent validity for this model was borderline, as AVE = 0.57.

### Composite reliability

Composite reliability of scores was adequate in the total sample for the past negative (ω = 0.84), past positive (ω = 0.66), present fatalistic (ω = 0.43), present hedonistic (ω = 0.61) and future focused (ω = 0.71).

**Table 2:** Items of the ZTPI in English and Factor Loadings Derived from the Confirmatory Factor Analysis (CFA) in the total sample

	Total
Factor 1: Past negative	
1	0.83
2	0.90
3	0.67
Factor 2: Past positive	
4	0.44
5	0.90
6	0.49
Factor 3: Present fatalistic	
7	0.26
8	0.51
9	0.52
Factor 4: Present hedonistic	
10	0.32
11	0.91
12	0.46
Factor 5: Future focused	
13	0.52
14	0.70
15	0.76

### Gender invariance

As reported in Table 3, all indices suggested that configural, metric, and scalar invariance was supported across gender. Given these results, we computed an independent-samples *t*-test to examine gender differences in ZTPI scores. The results showed that there was no statistically significant difference between men and women in all ZTPI dimensions. Higher mean past positive and future focused scores, as well as lower mean present hedonistic scores were significantly found in married people compared to single ones. Finally, a higher mean present fatalistic score was found in participants with a secondary level of education or less compared to those with a university education level (Table 4).

**Table 3:** Measurement Invariance of the ZTPI scale in the total sample

Model	χ²	*df*	CFI	RMSEA	SRMR	Model Comparison	Δχ²	ΔCFI	ΔRMSEA	ΔSRMR	Δ*df*	*p*
**Model 1: Across gender**												
Configural	274.52	158	0.915	0.042	0.078							
Metric	290.22	168	0.911	0.042	0.083	Configural vs. metric	15.7	0.004	< 0.001	0.005	10	0.108
Scalar	308.19	178	0.905	0.042	0.083	Metric vs. scalar	17.97	0.006	< 0.001	< 0.001	10	0.055
**Model 2: Across marital status**												
Configural	302.97	158	0.895	0.047	0.073							
Metric	315.58	168	0.893	0.046	0.074	Configural vs. metric	12.61	0.002	0.001	0.001	10	0.246
Scalar	326.34	178	0.892	0.044	0.074	Metric vs. scalar	10.76	0.001	0.002	< 0.001	10	0.376
**Model 1: Across education level**												
Configural	311.63	158	0.891	0.048	0.177							
Metric	334.44	168	0.882	0.049	0.181	Configural vs. metric	22.81	0.009	0.001	0.004	10	0.011
Scalar	340.91	178	0.884	0.047	0.181	Metric vs. scalar	6.47	0.002	0.002	< 0.001	10	0.774

*Note.* CFI = Comparative fit index; RMSEA = Steiger-Lind root mean square error of approximation; SRMR = Standardised root mean square residual

**Table 4:** Comparison between sexes in terms of the ZTPI scale and subscales scores in the total sample

	Past negative	Past positive	Present fatalistic	Present hedonistic	Future focused
**Gender**					
Males	9.85 ± 3.13	11.62 ± 2.52	8.50 ± 2.66	9.55 ± 2.48	11.92 ± 2.42
Females	10.07 ± 3.27	11.73 ± 2.48	8.39 ± 2.48	9.09 ± 2.54	12.05 ± 2.28
*p*	0.516	0.682	0.679	0.085	0.601
Effect size	0.068	0.044	0.042	0.183	0.055
**Marital status**					
Single	10.15 ± 3.18	11.51 ± 2.52	8.45 ± 2.46	9.43 ± 2.40	11.86 ± 2.31
Married	9.56 ± 3.30	12.25 ± 2.32	8.34 ± 2.76	8.69 ± 2.81	12.43 ± 2.31
*p*	0.097	**0.007**	0.711	**0.016**	**0.029**
Effect size	0.182	0.305	0.042	0.283	0.246
**Education level**					
Secondary or less	9.27 ± 3.84	11.86 ± 2.38	9.55 ± 3.07	8.59 ± 3.00	12.23 ± 2.25
University	10.04 ± 3.18	11.69 ± 2.50	8.36 ± 2.49	9.27 ± 2.50	11.99 ± 2.33
*p*	0.277	0.748	**0.032**	0.219	0.645
Effect size	0.218	0.070	0.425	0.246	0.104

Numbers in bold indicate significant *p* values

### Divergent validity

To assess the validity of the scores, we examined bivariate correlations with mental health issues in the present study using the total sample. Higher past negative and present fatalistic scores were significantly and positively correlated with higher depression, anxiety and stress. Higher past positive and future focused scores were significantly associated with less depression. Higher present hedonistic scores were significantly associated with more anxiety. Finally, older age was significantly associated with lower past negative, and present hedonistic scores and higher past positive scores (Table 5).

**Table 5:** Correlations of the ZTPI total scale and subscales scores with the other measures in the total sample

	1	2	3	4	5	6	7	8	9
1. Past negative	1								
2. Past positive	− 0.04	1							
3. Present fatalistic	0.21***	0.19***	1						
4. Present hedonistic	0.02	0.07	0.17***	1					
5. Future focused	− 0.11*	0.21***	− 0.13**	− 0.05	1				
6. Depression	0.32***	− 0.13**	0.25***	0.05	− 0.18***	1			
7. Anxiety	0.38***	− 0.06	0.20***	0.12*	− 0.09	0.71***	1		
8. Stress	0.33***	− 0.04	0.13**	0.07	− 0.07	0.63***	0.67***	1	
9. Age	− 0.10*	0.11*	0.07	− 0.15**	0.05	− 0.13**	− 0.16**	− 0.19***	1

*p < .05; **p < .01; ***p < .001

## Discussion

In the present study, we aimed to translate into Arabic and validate the shortened 15-item version of the “Gold standard” measure of time perspective, i.e. the ZPTI. Our findings suggest that the Arabic ZPTI-15 is internally consistent and psychometrically robust. As such, this brief and easy-to-use measure enables the conduction of future research in the field to purposively provide comprehensive insight into the time perspective patterns and correlates in Arab countries and the broad Arabic-speaking community globally.

CFA revealed that the five-factor model exhibited a good fit to the data, thus aligning with the original [1], other short forms’ validations [36, 42–44], as well as the English [45], Czech and Slovak [46] 15-item versions of the ZTPI [45, 46]. Besides, our findings demonstrated that the five ZTPI-15 subscales yielded a McDonald’ omega ranging from 0.43 to 0.84. Similarly, the validation study of the Czech and Slovak versions of the ZTPI-15 showed good internal consistency, with a Cronbach’s alpha varying from 0.65 to 0.78 [46]. Other previous translations also showed adequate internal consistency of the ZTPI as evidence by appropriate Cronbach’s alpha values (e.g., French (0.70–0.79) [24], Spanish (0.64–0.80) [25], Swedish (0.65–0.84) [32], and Lithuanian (0.63–0.79) [65]). We also found that multi-group CFA ascertained the invariance of the Arabic ZTPI-15 scale across gender at the configural, metric, and scalar levels; thus confirming that the 15 items were understood in the same way by our male and female participants. Consistently, gender invariance has previously been demonstrated with other short versions in different populations [47, 66]. These findings, therefore, suggest that the Arabic ZTPI-15 is recommended for future research on time perspective, and is useful for gender comparisons among Arabic-speaking people.

Our findings support divergent validity of the Arabic ZTPI-15 by showing positive correlations between past negative, present fatalistic, present hedonistic dimensions, and psychological distress; as well as negative correlations between past positive, future focused dimensions, and distress. These results were expected, and further confirm the findings of the validation study of the 15-item ZTPI [45] which demonstrated identical patterns of correlations with outcome variables to those of the long ZTPI. Having an increased sense of positivity towards the past predicts better psychological health and well-being [67] and enhances life satisfaction [45]; whereas endorsing a negative or unpleasant view of the past relates to greater psychological distress [1, 4, 68, 69] and reduced life satisfaction [70]. In line with our findings, previous studies also highlighted that both hedonistic and fatalistic present positively correlated with more severe depressive and anxiety symptoms [1, 67, 71]. In addition and similar to our results, Papastamatelou et al. found that Present Fatalistic and Past Negative orientations were linked to higher levels of perceived stress among Greek students [72]. Overall, negative attitude towards the past, hedonistic attitude towards life, and a hopeless/fatalistic view of present seem to be consistently involved in psychopathology and distress [17], while positive past and general future orientation seem to negatively predict positive psychological indicators [73].

In line with a previous study [74], our results showed that higher means past positive and future focused scores, as well as lower means present hedonistic scores were significantly found in married people compared to single ones, proving the Zimbardo’s (1999) hypothesis that married people with a positive experience in the past tend to see positive prospects for their future. Furthermore, marital status as a predictor of the past, present or future time perspective determines how the person perceives and reacts to the world. Thus, the family context in which the people are, in turn, determines expectations (positive or negative), as well as the readiness and skill to interact with the environment, to get to know their selves and to develop their abilities [74]. In addition, through the family, the continuity in the social development is ensured. The marriage is thought as a source of well-being that brings social and personal benefits in human life [75]; hence, marriage may itself lead to a surge in the expectations of each individual about their lives. This has been called the protective effect of marriage [76]. In other words, married people look forward to the future through the eyes of their children.

Our findings revealed that higher mean present fatalistic score was found in participants with a secondary level of education or less compared to those with a university education level, in line with a previous study [77]. Academic achievement was positively associated with positive attitudes toward the future and negatively associated with present fatalistic attitudes. In other words, individuals who achieved academically were more optimistic about their future and less pessimistic about their present than their less academically achieving counterparts.

### Study limitations

This study has some limitations that should be acknowledged. The first limitations lie to the self-report nature, cross-sectional design and recruitment method (online convenient sampling of non-clinical adults from Lebanon); which prevent causal inferences and generalization of our findings to the wider Arabic-speaking population. Future validations of the Arabic ZTPI-15 in clinical populations are still required. Another limitation consists of the fact that we did not assess other relevant psychometric properties of the ZTPI-15, such as test–retest reliability and predictive validity. Additional studies should consider addressing this limitation. In addition, while this study was able to confirm discriminant validity by correlation time perspective dimensions to depression, anxiety and stress, future studies still need to explore the patterns of correlations with other psychological and behavioral functioning (such as substance use, risky behaviors, coping strategies, and self-esteem). Information regarding the profession/occupation of the participants was not collected. Furthermore, given the method of recruitment, which was performed online, and mostly attracted educated and female participants, it is unlikely that our sample is representative of the wider Lebanese population. Consequently, the gender invariance results should be interpreted with caution because of the numbers inequality between males and females. Finally, while our sample comprises adults aged 65–85 years, we did not consider excluding persons with cognitive impairment, which could have influenced the results of our paper. Future studies should consider addressing this issue, while being aware that this may also decrease the clinical utility of research findings [78].

## Conclusion

We contribute the literature by providing clinicians and researchers with a brief, reliable and valid measure of the time perspective construct, the ZTPI-15. We believe that making available this psychometrically robust measure of the psychological dimension of time in the Arabic language will help foster cross-national and local research on this important construct in relation to various psychosocial and psychological indicators.

## Electronic supplementary material

Below is the link to the electronic supplementary material.

Supplementary Material 1 Arabic translation of the 15-item Zimbardo Time Perspective Inventory

## Data Availability

All data generated or analyzed during this study are not publicly available to maintain the privacy of the individuals’ identities. The dataset supporting the conclusions is available upon request to the corresponding author.
