# Supplementary material for: Psychometric properties of an Arabic translation of the briefest version of the Zimbardo time perspective inventory (ZTPI-15)
Source: BMC Psychiatry. 2023 May 12;23:338. doi: 10.1186/s12888-023-04815-8 (PMC10176805; doi:10.1186/s12888-023-04815-8)
Supplement: Supplementary file 1 — Supplementary Material 1 Arabic translation of the 15-item Zimbardo Time Perspective Inventory [file 12888_2023_4815_MOESM1_ESM.docx]

**Appendix 1. Arabic translation of the 15-item Zimbardo Time Perspective Inventory**

| غير صحيح | غير صحيح إلى حد ما | محايد | صحيح إلى حد ما | صحيح جدًا |  |
| --- | --- | --- | --- | --- | --- |
|  |  |  |  |  | أفكر في الأشياء السيّئة التي حدثت لي في الماضي |
|  |  |  |  |  | التجارب السابقة المؤلمة تعود الى ذهني |
|  |  |  |  |  | من الصعب عليّ أن أنسى صور غير سارة من فترة الشباب |
|  |  |  |  |  | غالبًا ما تعيد الصور والروائح والاصوات المألوفة في طفولتي طوفانًا من الذكريات الرائعة |
|  |  |  |  |  | الذكريات السعيدة للأوقات الممتعة تحضر إلى ذهني بسهولة |
|  |  |  |  |  | أستمتع بقصص حول كيف كانت الأمور في الماضي الجميل |
|  |  |  |  |  | الحياة في الوقت الحاضر معقّدة كثيراً، أنا أفضّل حياة الماضي البسيطة |
|  |  |  |  |  | نظرًا لأن كل ما سيكون سيكون، لا يهم حقًا ما أفعله |
|  |  |  |  |  | غالبًا ما يؤتي الحظ ثماره أفضل من العمل الشاق |
|  |  |  |  |  | أتخذ قرارات إرتجالاً |
|  |  |  |  |  | المخاطرة تمنع حياتي من أن تصبح مملة |
|  |  |  |  |  | من المهم أن أضع الإثارة في حياتي |
|  |  |  |  |  | عندما أريد إنجاز شيء، أضع أهدافًا وأخذ بعين الاعتبار وسائل محددة للوصول إلى تلك الأهداف |
|  |  |  |  |  | يأتي الالتزام بمواعيد الغد النهائية، وإنهاء جميع الأعمال الضرورية، قبل قضاء ليلة مُسَلِّية |
|  |  |  |  |  | أكمل المشاريع في الوقت المحدد بإحراز تقدّم مستمر |
